# Digital therapeutics for mild adolescent idiopathic scoliosis: a protocol for a randomized controlled trial

**DOI:** 10.1186/s12887-026-07457-3

**Published:** 2026-08-15

**Authors:** Yingxian Li, Xuan Zhou, Xiaoqing Zhu, Xiaoyan Yang, Xin Li, Mengdie Jin, Hong Yu, Qimeng Fan, Hong Wang, Qing Du

**Affiliations:** 1 Department of Rehabilitation Medicine, Xinhua Hospital, Shanghai Jiao Tong University School of Medicine, Shanghai, 200092 China; 2 Department of Rehabilitation Medicine, Huadong Hospital, Fudan University, Shanghai, China; 3 College of Rehabilitation Sciences, Shanghai University of Medicine & Health Sciences, Shanghai, 200237 China; 4 Children’s Rehabilitation Innovation and Transformation Research Center of Yuanshen Rehabilitation Institute, Shanghai Jiao Tong University School of Medicine, Shanghai, 200025 China

**Keywords:** Idiopathic scoliosis, Digital therapeutics, Cobb angle, Fall index, Pulmonary function, Quality of life

## Abstract

**Background:**

Digital therapeutics (DTx), a new type of intervention presented in the last few years, can be used to provide patients with access to evidence-based therapeutic interventions using high-quality software programs to achieve the purpose of treating, managing, or preventing diseases. The purpose of this study is to explore the efficacy of home-based digital therapy in treating mild adolescent idiopathic scoliosis (AIS).

**Methods:**

This study will be a prospective, single-blind, randomized controlled, two-arm parallel-group, confirmatory superiority clinical trial. The subjects will include 106 patients aged 10 to 17 years with mild AIS and a major Cobb angle of 10° to 20°. Subjects will be randomly assigned to either a digital therapy group (DTx) or a control group (conventional rehabilitation) for 12 months of treatment. Assessments occur at baseline, 6, and 12 months. Radiographic outcomes will be independently evaluated on anonymized images. The primary outcome is the major Cobb angle. Secondary outcomes are the fall index (FI), forced vital capacity (FVC), and Scoliosis Research Society-22 (SRS-22) score. Data will be analysed using linear mixed-effects models (LMM).

**Discussion:**

The results of this confirmatory superiority trial will show whether DTx has superior effects on improving spinal deformity, balance function, pulmonary function, and quality of life compared with conventional rehabilitation in patients with mild AIS.

**Trial registration:**

The trial was registered prospectively at www.chictr.org.cn on September 25th, 2023 (ChiCTR2300076105).

**Supplementary Information:**

The online version contains supplementary material available at https://doi.org/10.1186/s12887-026-07457-3.

## Background

Scoliosis is defined as a three-dimensional spinal malformation characterized by a lateral curvature of one or more spinal segments in the coronal plane ≥ 10°, accompanied by rotation of the spine in the transverse plane and changes in the sagittal plane alignment [1]. A Cobb angle between 10° and 20° is considered mild scoliosis. Adolescent idiopathic scoliosis (AIS) represents the most common type, accounting for 80% of idiopathic cases. Since adolescence is a period of rapid spinal growth, a significant proportion of AIS patients, up to two-thirds according to recent epidemiological studies, experience deformity progression during this period [2]. Without timely intervention, progressive malformations can affect the appearance of the trunk, cause mental health problems, and negatively impact biomechanical stability [3–5]. 

While severe cardiopulmonary compromise is typically associated with curves exceeding 50°, recent evidence indicates that even mild AIS can induce early biomechanical alterations in thoracic mobility and subclinical deficits in sensorimotor integration and postural control. Therefore, evaluating forced vital capacity (FVC) and balance stability in mild AIS is clinically relevant for establishing functional baselines and comprehensively assessing the holistic impact of early interventions. Specifically, the Tetrax posturography system, which has been validated for assessing postural stability in pediatric and adolescent populations, provides a reliable fall index (FI) to quantify these early balance deficits [6]. 

To prevent the progression of spinal deformity, the SOSORT guidelines recommend physiotherapy scoliosis-specific exercises (PSSEs) as the preferred treatment for mild AIS [1, 7]. Although the efficacy of PSSEs is well-confirmed, adolescents are in a critical period of growth and development. Demanding schoolwork and the subtle, asymptomatic nature of mild scoliosis often lead to poor treatment compliance [8, 9]. The time and economic costs of regular hospital visits further limit the feasibility of long-term conventional rehabilitation.

Digital therapeutics (DTx) are a new type of evidence-based intervention delivered through high-quality software programs to treat, manage, or prevent diseases [10]. By transcending the spatial and temporal limitations of traditional outpatient care, DTx provides a convenient and cost-effective alternative. It enables patients to obtain remote rehabilitation guidance, real-time feedback, and continuous quality monitoring, thereby improving medical resource allocation and patient adherence.

Currently, there is a lack of high-quality application of digital therapies in the field of scoliosis. Therefore, the objective of this study is to determine whether a digital therapy program provides superior improvements in spinal deformity, balance function, pulmonary function, and quality of life compared with conventional rehabilitation among adolescents with mild AIS.

## Methods

### Study design

**Fig. 1:**
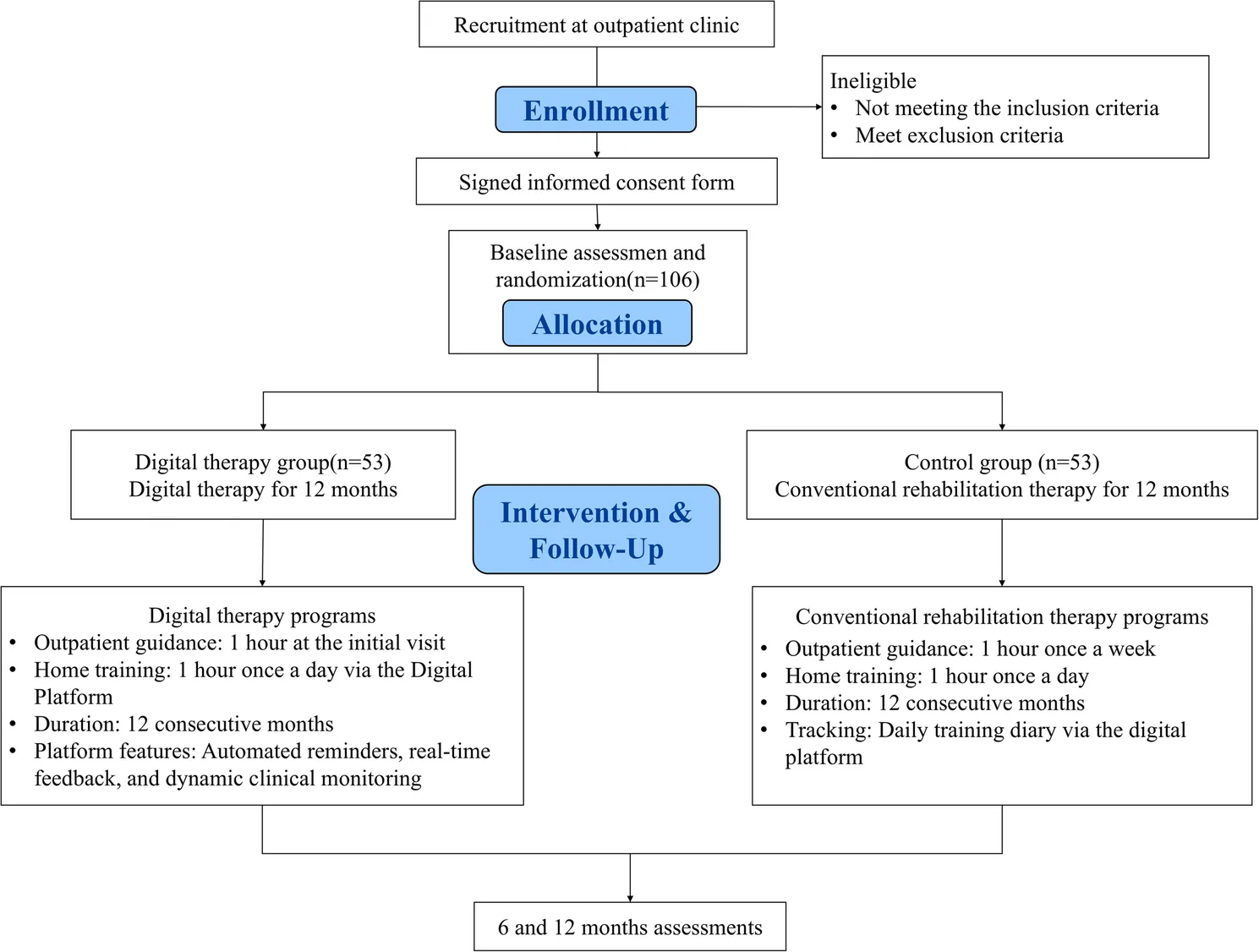
Study participation flowchart

This study will adopt a prospective, single-blind, randomized controlled, two-arm parallel-group, confirmatory superiority clinical study design (Fig. 1) and refer to the Food and Drug Administratin (FDA) certification management standards for digital therapy to carry out clinical trials of digital therapy for patients with mild AIS [11]. This trial will validate the management efficacy of this model.

### Participants

Patients with the following will be enrolled in the study: patients who meet the diagnostic criteria for idiopathic scoliosis; [1] patients aged 10 to 17 years; patients who have a Cobb angle of 10° to 20° on the primary curve based on X-ray findings; patients who are skeletally immature with Risser’s sign grades I-IV; patients who can learn and operate the cell phone program; and patients who willingly participated therein and signed a documented informed consent form agreeing to the requirements of the trial. The following patients will be excluded: patients with functional scoliosis (including bilateral lower extremity inequality, hip contracture, inflammatory stimuli, etc.); patients with congenital or neurological scoliosis or scoliosis caused by other diseases; patients with a history of neurological, neuromuscular, cardiovascular, and other systemic disorders; patients with a history of spinal rehabilitation or bracing; and patients with contraindications to exercise who are unable to tolerate exercise therapy. The study was ethical committee-approved and prospectively registry-approved at www.chictr.org.cn (ChiCTR2300076105).

### Recruitment

106 patients will be recruited in the outpatient clinic of the Department of Rehabilitation Medicine, Xinhua Hospital Affiliated to Shanghai Jiao Tong University School of Medicine, and there will be informed consent forms signed by the patients and their guardians.

### Randomization

There will be a 1:1 allocation of subjects to digital therapy group and control group. Randomization information (random seed 16264) will be generated directly with the use of the randomization website (www.randomization.com), and the resulting assignment information will be sealed, placed in opaque-sealed envelopes with codes written on the outside, and then handed to the investigator. The subjects will be numbered once they enter the study, and the corresponding numbered envelopes will not be opened until all baseline assessments have been completed. The intervention will be delivered in an envelope fashion. The coders will not participate in the recruitment, intervention, or evaluation of the trial subjects. As this study will be an open study, physicians, therapists, and patients cannot be blinded while providing or receiving treatment. Nevertheless, it will be requested that patients do not disclose their subgroups to ensure that all assessors are blinded to the treatment. To maintain strict blinding, these independent, trained assessors will conduct all functional and physiological evaluations in a dedicated testing room separate from the treatment area. They will complete the assessment of all subjects before initial treatment, after 6 months and 12 months of the intervention. Specifically for radiographic outcomes, assessor blinding will be strictly preserved by having independent personnel perform the measurements on the anonymized images, without any contact with the subjects or the intervention process.

### Interventions

#### Digital therapy group

##### Scoliosis digital management platform

A physician will assist all subjects in constructing a personal electronic health record on the Scoliosis Digital Management Platform. This platform is developed as a WeChat applet with a dual-end architecture (a patient-facing application and a physician-facing monitoring dashboard). Unlike simple telerehabilitation systems that merely provide educational content, our platform integrates automated exercise reminders, structured instructional videos, and an automated feedback mechanism. The first spinal assessment questionnaire developed in-house will be used immediately after enrolment to conduct a baseline survey, including demographic information and disease information; disease information will be obtained from the medical records of the hospital’s information system, which includes body mass index (BMI), sitting height, data on menstrual cycles, family history, and previous exercise, etc. Finally, the data will be integrated, and a personalized management program will be provided for each patient based on the results of the integration. Therapeutic decisions and exercise progressions are generated by the clinical team based on real-time data uploaded to the physician’s dashboard.

##### Development of a personalized digital management program

**Fig. 2:**
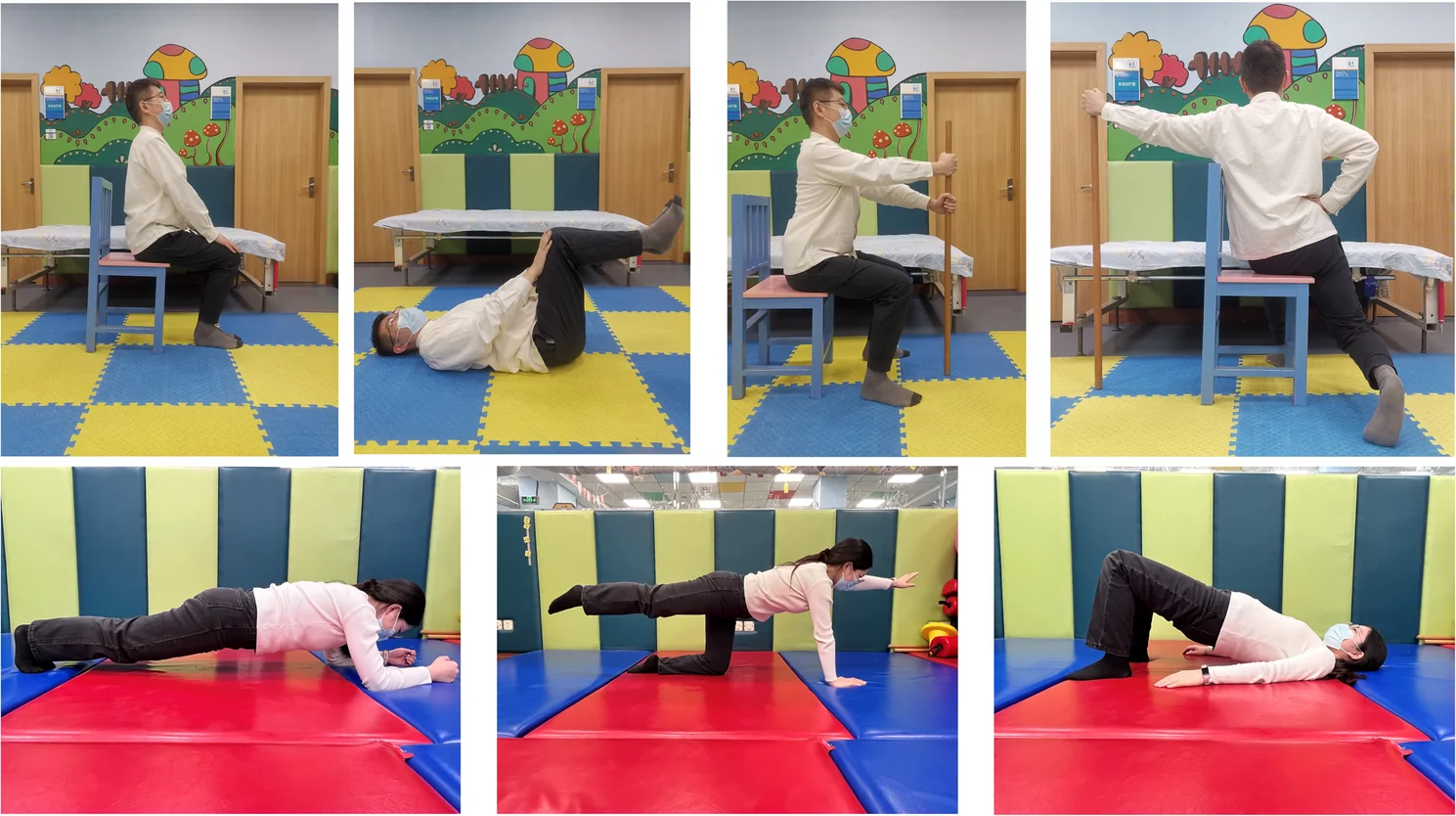
Examples of conventional rehabilitation therapy for scoliosis

**Fig. 3:**
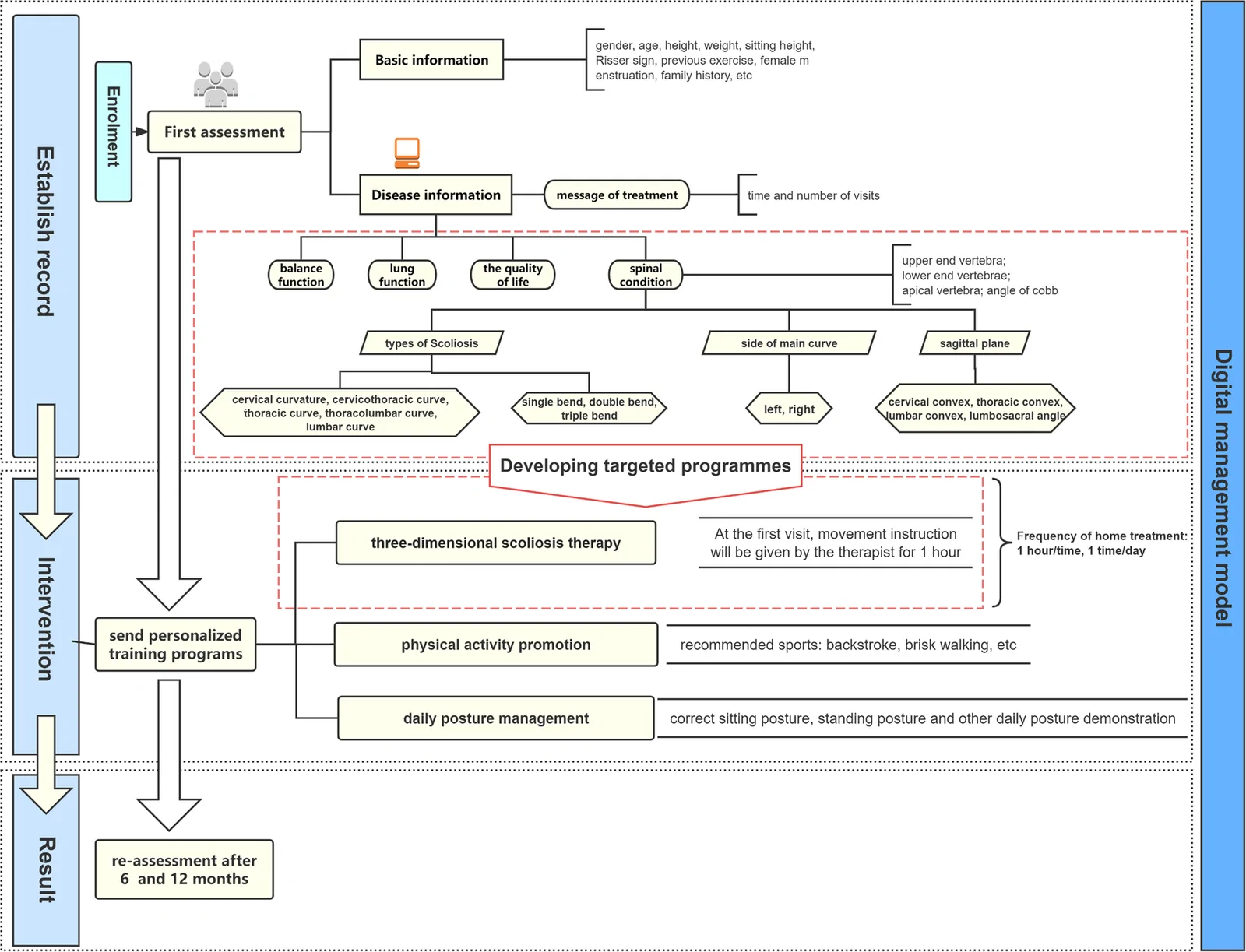
Digital management model flowchart

**Fig. 4:**
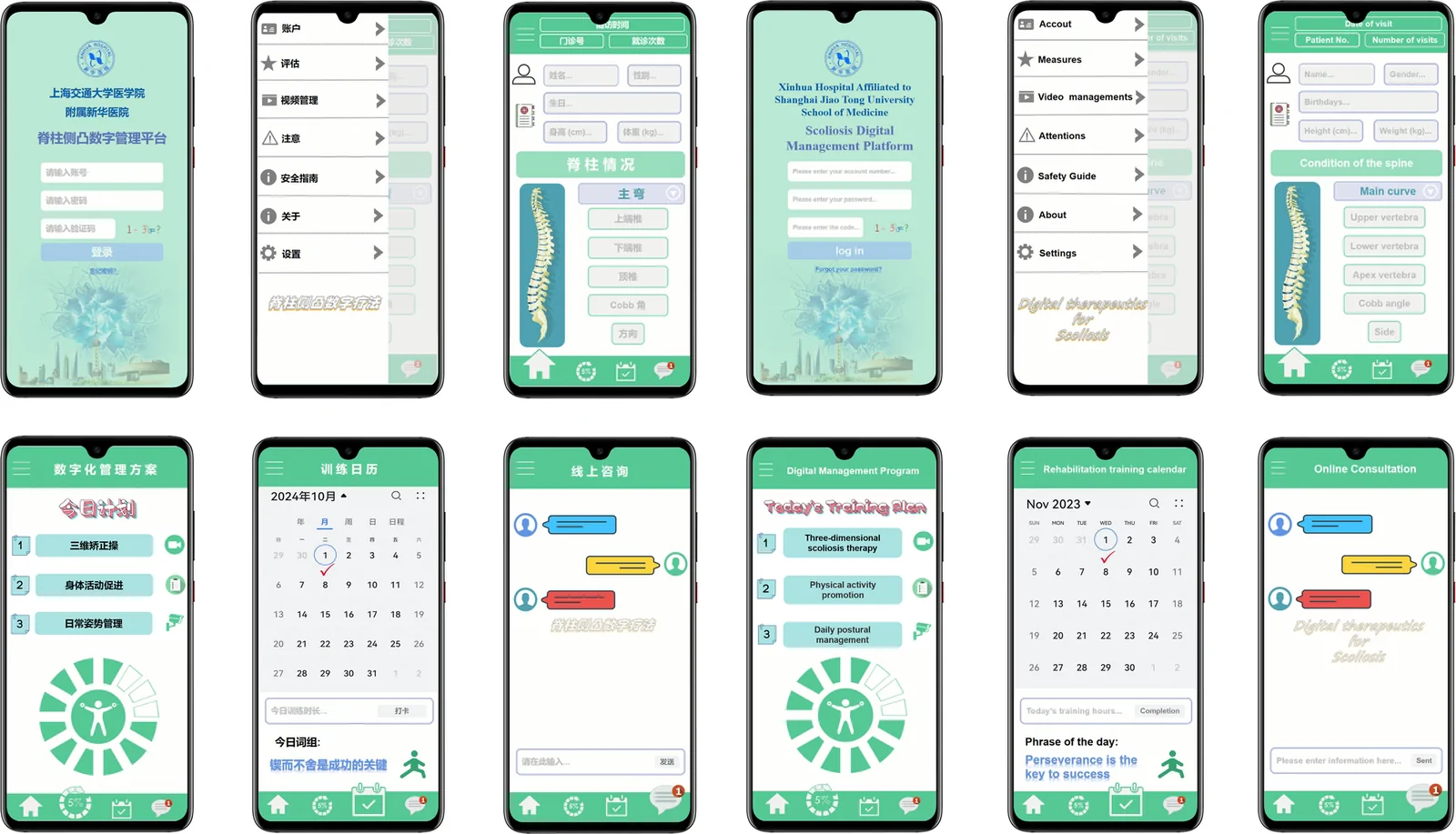
Examples of digital therapeutic app screens (Chinese and English versions)

Subjects will receive 1 h of movement instruction by a rehabilitation therapist at the initial visit, including three-dimensional scoliosis therapy, physical activity promotion, and daily postural management (some examples are shown in Fig. 2) [12]. Through a homemade digital management system, we will provide digital rehabilitation strategies and guidance for different patients with specific spinal deformity conditions, perform spinal assessments of the emergence of dysfunctions, evaluate daily living habits, and perform real-time quality monitoring of the rehabilitation treatment. The intensity of treatment will be set according to each individual’s fatigue level, and the treatment will be provided at home in 1-hour sessions once per day for 12 consecutive months. The rationale for this daily 1-hour prescription is to leverage the inherent advantages of digital therapeutics, which aim to overcome the spatial and temporal barriers of traditional outpatient visits, thereby increasing the patient’s training exposure and treatment adherence. Details are shown in Figs. 3 and 4.

#### Control group

The control group will receive conventional rehabilitation therapy, with 1 h of movement teaching by one of the same rehabilitation therapists in the digital therapy group at the initial visit, including three-dimensional scoliosis therapy, physical activity promotion, and daily postural management, with the intensity of the treatment set according to the level of fatigue of each individual. Treatment will be provided at the hospital outpatient clinic in 1-hour sessions once per week and performed once daily for 60 min at home. To ensure consistent data collection, control subjects will also use the digital management platform to record their daily home exercise sessions in a digital training diary. Continuous treatment will be provided for 12 months.

### Outcomes

Subjects participating in the trial will be required to undergo three comprehensive assessments: assessments at the initial visit, after 6 months and 12 months of the intervention. Specific assessments will include demographic and physical developmental indicators of the subjects (including sex, age, height, weight, BMI, and Risser’s sign grades), balance function (FI), X-ray measurement (Cobb angle of the main curve), FVC and quality of life (Scoliosis Research Society-22 items questionnaire). Assessors will be blinded to group assignments and collect all outcome measures at the initial visit, after 6 months and 12 months of the intervention.

### Participant timeline

**Fig. 5:**
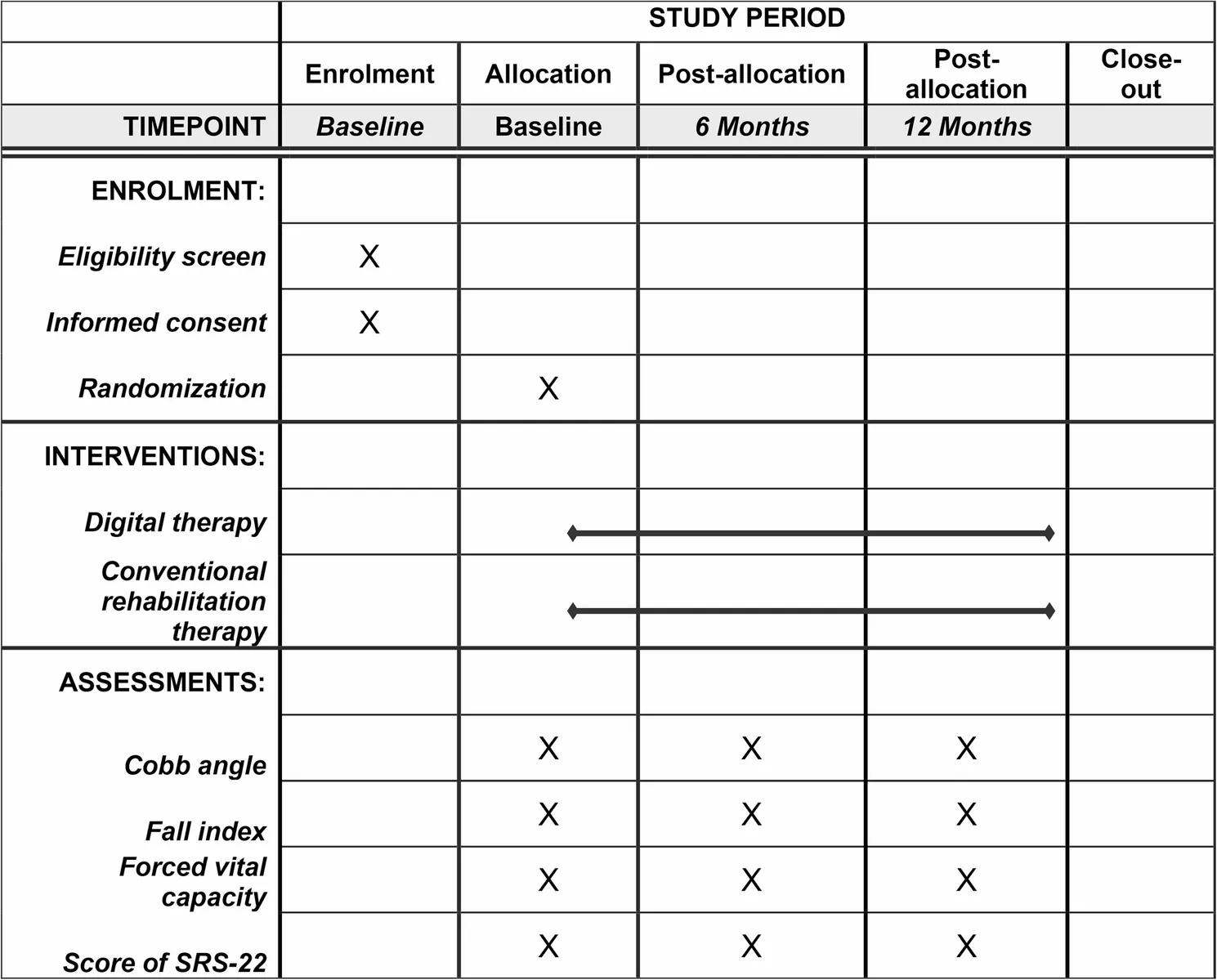
Standard protocol items: Recommendations for interventional trials (SPIRIT) figure

The participant timeline, including the schedule of enrolment, interventions, and assessments, is summarized in the SPIRIT flowchart (Fig. 5). Briefly, after eligibility screening and obtaining informed consent, all participants will undergo comprehensive baseline assessments. Following baseline data collection, participants will be randomly allocated to either the digital therapy group or the control group. Both groups will receive continuous interventions for 12 months. To dynamically monitor the therapeutic effects, the complete set of outcome measures—including the major Cobb angle, balance function (FI), pulmonary function (FVC), and quality of life (SRS-22)—will be collected at three specific time points: baseline (pre-allocation), 6 months post-intervention, and 12 months post-intervention. Finally, trial close-out procedures will be completed following the final 12-month assessments.

### Primary Outcome

#### Change in the Cobb angle of the major curve

The Cobb angle of the main curve will be measured on full-spine radiographs using the Cobb method [13]. To minimize measurement bias, all radiographic images will be strictly anonymized before measurement to mask patient identity and subgroup allocation. The images will then be independently evaluated by two trained assessors who are completely blinded to the trial design. To ensure measurement reliability, intra-class correlation coefficients (ICC) will be calculated to assess both intra-rater and inter-rater reliability for the Cobb angle measurements.

### Secondary Outcome

#### Fall index

A static balance metre (Tetrax°) (Sunlight Medical Ltd, Israel) will be used to test the balance function of the subjects. The intragroup correlation coefficient for this testing method is 0.86, showing good retest reliability [6]. During the measurement, the patient will be required to maintain eight positions on the tester, which are standing on the force platform with eyes open or closed, standing on the foot cushion with eyes open or closed, turning the head to the left or right with eyes closed, and tilting the head forward or backwards by 30° with eyes closed. The trained assessors will measure balance function indicators. According to the indicators, i.e. the FI, the balance function of the subject will be judged, and the fall risk will be clarified [14]. 

#### Forced vital capacity

Currently, the most commonly used index for evaluating pulmonary function is FVC. Pulmonary function tests will be performed using the QuarkPFT4 spirometer (COSMED, Italy) [15]. When the test begins, the subject will take a standing position and hold the disposable cartridge blowpipe with his or her lips. The trained assessor will follow the instructions of the instrument and instruct the subject to breathe calmly first, ensuring that the screen displays at least 5 stable tidal breaths; then, the examiner will instruct the subject to perform a maximal inhalation up to the total lung volume, exhale as fast as he or she can up to the residual volume level, and inhale up to the total lung volume level, finally obtaining a measurement result. The test will usually be repeated three times, and the average of the three measurements will be taken.

#### The scoliosis research society-22 item questionnaire scores

The Scoliosis Research Society-22 item questionnaire (SRS-22) is a simple, practical health-related scale for IS that is recommended globally by the Scoliosis Research Society and is widely used to assess the impact and efficacy of scoliosis. The SRS-22 consists of 22 items covering 5 dimensions, each with a score ranging from 1 to 5, including functional activity, pain, self-image, mental health, and satisfaction with treatment, with higher scores indicating a better quality of life for the patient [16]. 

### Compliance supervision

The personnel involved in the study are qualified, and the division of responsibilities will be clearly defined before the study begins. Personnel involved in data collection and data entry will be trained and pass the examination, and therapists and digital therapy supervisors will also be trained and pass the examination to ensure homogenization.

To ensure a fair and objective evaluation, a standardized compliance quantification formula will be applied to both groups. Overall compliance will be defined as the percentage of completed prescribed exercise sessions out of the total expected prescribed sessions over the 12-month period. For the digital therapy group, compliance data will be objectively and automatically extracted from the backend server logs of the spine health digital management platform (WeChat applet), and subjects will clock in daily. As an inherent mechanism of the digital therapeutics to promote adherence, subjects will receive the appropriate free mission content and virtual/substantial rewards based on the number of times they clock in.

For the control group, compliance will be monitored using a combination of a standard family training diary and hospital attendance logs. Parents will record the time and frequency of the daily 30-minute self-corrective home exercises in the diary. Additionally, therapists will maintain strict clinic attendance logs for the weekly 1-hour supervised sessions. The quality of self-corrective gymnastics movements will be controlled weekly by the therapist through on-site audits while the control subjects are at the health care facility.

Quality control personnel will regularly check the patient’s daily training through this platform. Subjects who complete the study will receive an additional commemorative award and a free follow-up program instruction course. At the end of the trial, the quantified compliance rates (percentage of completed sessions) will be statistically compared between the digital therapy and control groups using a chi-square test or independent t-test to evaluate the impact of the digital platform on treatment adherence.

### Adverse events

In the trial period, the physiotherapist will faithfully record all adverse events in an adverse event form. The researchers will assess the association between the adverse events and the intervention and decide whether to discontinue the intervention. Meanwhile, the adverse events will be immediately targeted and followed up until the symptoms disappear.

### Radiation safety considerations

Given that Cobb angle measurements require repeated radiographs, strict radiation safety protocols will be implemented. The imaging frequency (at baseline, 6 months, and 12 months) strictly adheres to the established international guidelines for monitoring mild adolescent idiopathic scoliosis [1]. To further minimize radiation exposure for the adolescent participants, standard low-dose digital radiography protocols or low-dose imaging systems (e.g., the EOS imaging system) will be utilized for all radiographic examinations.

### Data analysis

The data analysis set will follow the intention-to-treat (ITT) analysis principle, and all individuals participating in randomization will be included in the study analysis set for the primary and secondary indicators. Missing data will be handled internally using linear mixed-effects models (LMM) based on maximum likelihood estimation. The modified ITT (mITT) set for the primary and secondary outcome indicators in this study will include all subjects who participate in the randomized subgroups and at least one intervention. In addition, the per-protocol (PP) set will be used to supplement the mITT set. The PP set will include subjects who perform the trial procedure in strict compliance with the protocol. The safety analysis set (SS) will include all subjects who receive at least one intervention. The mITT set will be used for both primary outcome indicators and secondary outcome indicators, with the PP set as a supplement. The mITT set will be used for both primary outcome indicators (Cobb angle of the main curve) and secondary outcome indicators (FI, FVC, and SRS-22 score), with the PP set as a supplement.

Data will be entered using Epidata 3.0 (Odense, Denmark) and analysed using Stata 14.0 statistical software. The Kolmogorov‒Smirnov test or Shapiro-Wilk test will be used on the variables to test if they conform to a normal distribution. For baseline characteristics, normally distributed continuous variables will be expressed as the mean±standard deviation $\left(\overline x\pm s\right)$, and comparisons between groups will be made using the independent samples t-test. When the data distribution is abnormal, the median (M) and quartiles (P25, P75) will be used. Comparisons between groups will be made using the Mann-Whitney U test. Rank data will be compared using the Wilcoxon rank sum test. Count figures will be shown as a percentage of the cases, and a chi-square test will be used to compare the groups.

To analyze the longitudinal data across the three measurement points (baseline, 6 months, and 12 months), LMM will be utilized to evaluate the time × group interactions. The LMM will include group, time, and the group-by-time interaction as fixed effects, and the subjects as a random effect. To control for the difference in treatment intensity between the two groups, the total exercise dose will be included as a covariate in the model. The primary statistical comparison will focus on the between-group difference in the change of the major Cobb angle from baseline to 12 months. For post-hoc comparisons across the three time points to control for multiple testing, a Bonferroni correction will be applied, setting the significance level at *P* < 0.017 (0.05/3).

### Sample size calculation

G*Power 3.1.9.7 (Heinrich-Heine-Universität Düsseldorf, Germany) software was used for sample size calculation. The primary endpoint for this study is the Cobb angle of the main curve. According to the results of the relevant study by Küçük et al. [17], the post-treatment mean Cobb angles were 6.8° ± 3.71° in the experimental group and 10.0° ± 6.87° in the control group. Based on these values, the expected between-group effect size at the final 12-month follow-up was calculated as 0.58. Although this study involves longitudinal repeated measurements, the sample size calculation was performed conservatively based on the final between-group difference to ensure sufficient power for the primary comparison. With α set to 0.05 and β set to 0.20, 48 patients need to be enrolled in each group. Taking into account a possible lost-to-follow-up rate of 10%, 53 patients need to be enrolled in each group, resulting in a total planned enrolment of 106 patients.

## Discussion

One potential confounding factor in this trial design is the difference in intervention dose between the two groups. The digital therapy group will perform 1-hour sessions daily, whereas the control group will receive weekly 1-hour supervised sessions combined with 30-minute daily home exercises. This difference in treatment intensity is designed to reflect the real-world advantages of digital therapeutics, which inherently aim to increase overall training exposure and compliance compared to traditional care. We will consider this difference in treatment intensity as a potential confounder during the statistical analysis.

Digital therapy has gradually become a new treatment option for rehabilitation. This randomized controlled clinical study will establish a digital management model for patients with mild AIS and compare its 12-month efficacy against conventional rehabilitation. The trial will evaluate whether digital therapy provides greater improvements in spinal deformity, balance function, pulmonary function, and quality of life. If the results show superior clinical outcomes, this study will provide high-quality evidence for digital telerehabilitation. Furthermore, effective digital therapy could offer a more accessible treatment option for school-aged children, reduce the time and financial burden on their families, and help decrease the overall pressure on the health system.

## Supplementary Information

Supplementary Material 1.

## Data Availability

No datasets were generated or analysed during the current study.

## Abbreviations

AIS: Adolescent idiopathic scoliosis
FVC: Forced vital capacity
PSSEs: Physiotherapy scoliosis-specific exercises
DTx: Digital therapeutics
FDA: Food and Drug Administration
BMI: Body mass index
FI: Fall index
ICC: Intra-class correlation coefficients
SRS-22: The Scoliosis Research Society-22 item questionnaire
ITT: Intention-to-treat
LMM: Mixed-effects models
mITT: Modified ITT
PP: Per-protocol
SS: Analysis set
